# Dental Anxiety among Medical and Paramedical Undergraduate Students of Malaysia

**DOI:** 10.1155/2017/4762576

**Published:** 2017-02-28

**Authors:** Shilpa Gunjal, Deepak Gowda Sadashivappa Pateel, Sujal Parkar

**Affiliations:** ^1^Department of Dental Public Health, Faculty of Dentistry, MAHSA University, Bandar Saujana Putra, 41200 Jenjarom, Selangor, Malaysia; ^2^Department of Oral Pathology and Oral Medicine, Faculty of Dentistry, MAHSA University, Bandar Saujana Putra, 41200 Jenjarom, Selangor, Malaysia; ^3^Department of Public Health Dentistry, Siddhpur Dental College and Hospital, Siddhpur, Patan, Gujarat, India

## Abstract

*Aim*. To assess the dental anxiety level among dental, medical, and pharmacy students of MAHSA University, Malaysia.* Materials and Methods*. A cross-sectional questionnaire study was conducted among 1500 undergraduate students of MAHSA University. The Modified Dental Anxiety Scale (MDAS) was used to measure dental anxiety among the study population. The responses were assessed by 5-point likert scale ranging from 1 to 5. The level of anxiety was categorized into lowly anxious (5–11), moderately anxious (12–18), and severely anxious ≥19. Out of 1500 students enrolled, 1024 students (342 males and 682 females) completed and returned the questionnaire having response rate of 68.26%.* Results*. There was a statistically significant difference (*P* < 0.001) when the mean dental anxiety scores were compared among the three faculties and dental students had lowest mean score (11.95 ± 4.21). The fifth year (senior) dental students scored significantly (*P* = 0.02) lower mean anxiety score as compared to the first dental students (junior). The students were anxious mostly about tooth drilling and local anesthetic injection.* Conclusions*. Dental students have a significantly low level of dental anxiety as compared with medical and pharmacy students. Incorporation of dental health education in preuniversity and other nondental university curriculums may reduce dental anxiety among the students.

## 1. Introduction

In the field of behavior science, the terms dental fear and anxiety are highly related and often used interchangeably in the literature. A distinction between “normal” dental fear and “pathologic” dental anxiety must therefore be made. Normal fear is a physiological, behavioral, and emotional response to a feared object or situation [1]. Pathological anxiety is characterized by the loss of the original signaling function of the anxious response, which can be triggered by objectively harmless situations. It is considered too strong and persistent in relation to the dangerous stimulus, or it is related to an unreasonable future threat [2].

In the last 2-3 decades, dentistry has made recent advances in the curative as well as in the preventive aspect for providing better dental care to the patients. Yet these advances have not been able to eradicate or markedly diminish dental fear and anxiety among patients. Dental anxiety might affect dentist-patient relationship, resulting in ambiguous diagnosis of the genuine dental problem and delay in the dental treatment by the patients which results in deterioration of their oral health [3, 4].

Anxiety related to dental treatment is not only puzzling for patients, but it is also a major source of worry for dental practitioners to treat anxious patients. There is now ample evidence suggesting that the physiological stress indicators like increased blood pressure, elevated heart rate, and so on which dentist experiences are equal to the responses of the patients when procedures are being performed [5]. One important group is the university students, who are future healthcare providers. Therefore, it is necessary for these students to learn about the techniques that can help them to overcome their own dental anxiety [6]. A decrease in their anxiety levels would make them confident clinicians, who could in turn treat their patients well.

Through the extensive literature review it was revealed that the prevalence of dental anxiety ranged from 11% to 27.5% among the undergraduate students of various universities throughout the globe [7–9]. However, presently only one study is reported among Malaysian population [10]. Hence, the present study was carried out to assess the dental anxiety levels among dental, medical, and pharmacy undergraduate students of MAHSA University, Malaysia.

## 2. Material and Methods

### 2.1. Study Design

A descriptive, cross-sectional questionnaire study was conducted among dental, medical, and pharmacy undergraduate students of MAHSA University, Malaysia. Prior to conducting the study, the protocol of the study was submitted to Research Review Committee of MAHSA University and ethical approval was obtained to conduct the study. A total of 1500 students were enrolled in this study on a voluntary basis, and an informed consent was obtained from all students. Both male and female students were included in the study.

### 2.2. Questionnaire Used in the Study

Anxiety related to dental treatment was assessed by means of Corah's Dental Anxiety Scale (CDAS) [11]. The parameters of the scale include anticipation of treatment the next day, sitting in waiting room, getting a tooth drilled, and getting teeth scaled or polished. The CDAS unfortunately does not enquire about local anesthetic injection, which is a focus for some patients' anxiety [12]. Hence the Modified Dental Anxiety Scale (MDAS) was used where an extra item has been included referring to the respondent's feelings towards local anesthetic injection with especial reference to the site of the injection [13]. MDAS included 5 questions, in which each question had five responses (5-point likert scale) ranging from not anxious, slightly anxious, and very anxious to extremely anxious. The scores for each of the 5 questions were summed to give the level of dental anxiety. The overall maximum score is 25. Based on this the anxiety level was categorized into lowly anxious (5–11), moderately anxious (12–18), and severely anxious ≥19.

### 2.3. Data Collection

Students were informed about the objectives of the study before administration of questionnaire. They were also informed that no incentives will be provided for the participation of the study. Questionnaire was distributed during leisure time and participants were given sufficient time to complete them. To determine the test-retest reliability of the questions, 30 students who completed the study during initial administration completed the survey after 15 days. Confidentiality of the data was maintained.

### 2.4. Statistical Analysis

The reliability of the questionnaire was assessed using Cronbach's alpha coefficient. Those questionnaires which were completed were considered for the analysis. The data were analyzed by applying descriptive and inferential statistical analysis as and when appropriate. Chi-square test and Kruskal-Wallis tests were used to compare the qualitative variables, while one-way ANOVA was used to compare the quantitative variables. Analysis was carried out using Statistical Package for Social Science (SPSS) version 17 (SPSS Inc., Chicago, IL, USA). The level of significance was kept at 5%.

## 3. Results

Out of 1500 students enrolled, 1024 students completed and returned the questionnaire, which accounts for a response rate of 68.26%. Cronbach's alpha coefficient value was found to be 0.86, suggesting good internal consistency. Among 1024 undergraduate students, there were 342 males and 682 female students. The age of the study population ranges from 18 to 29 years having mean age of 22.15 ± 1.73 years. The study subjects included dental (*n* = 308; 30.08%), medical (*n* = 542; 52.93%), and pharmacy (*n* = 174; 16.99%) undergraduate students. The distribution of the study subjects according to the faculty, age, and gender is demonstrated in Table 1.

Table 2 depicts the faculty-wise distribution of study subjects by the level of dental anxiety. Out of 1024 students, 43.16% (*n* = 442) of the students had moderate level of anxiety. The high level of anxiety was found among 10.35% of medical students as compared to 2.15% for dental students. The difference was found to be statistically significant (*P* < 0.001). The mean score for dental students was low (11.95 ± 4.21), while that of pharmacy students was high (14.49 ± 5.09). There was a statistically significant difference (*P* < 0.001) when the mean anxiety scores were compared among three faculties.

Year-wise comparison of dental anxiety among dental students also revealed statistically significant difference (*P* = 0.02). However, the year-wise comparison of dental anxiety between medical and pharmacy students showed no statistically significant difference (*P* > 0.05) (Table 3).

The year-wise comparison of mean dental anxiety scores for all the three faculties was made (Table 4). The mean score of anxiety among dental and medical students significantly (*P* = 0.02) decreases while for pharmacy students the score significantly increases (*P* = 0.02).

The mean scores of the items were compared among all the three faculties (Table 5). The dental students showed lower score which was statistically significant (*P* < 0.001) as compared with medical and pharmacy students.

## 4. Discussion

Dental anxiety is one of the major concerns in the routine clinical practice that should be dealt with and managed for the proper oral healthcare. Therefore, the present study was conducted to investigate the dental anxiety among undergraduate students of MAHSA University, Malaysia, by using MDAS questionnaire [13]. The original Corah's Dental Anxiety Scale (CDAS) [11] has been widely used in dental research. However, the modified version introduced by Humphris et al. [13] is valid, reliable, brief, accessible, and easy to use; thus, it was used to assess the level of dental anxiety in the present study. The Cronbach alpha coefficient of MDAS in this study was 0.86. This was in accordance with previous studies conducted by Serra-Negra et al. [7], Marginean and Filimon [14], Tunc et al. [15], and Humphris et al. [16] whose Cronbach alpha coefficient was 0.80, 0.90, 0.91, and 0.95, respectively.

In the present study, the mean dental anxiety score for dental students was significantly lower compared to medical students, while it was higher for pharmacy students. The reason for higher anxiety scores in pharmacy students compared to medical students may be due to lack of dental awareness education among pharmacy students, while the medical students are supposed to be more familiar with stress management related to health measures. This result was in line with previous studies done by Cesar et al. [9], Sghaireen et al. [17], and Abu Hantash et al. [18]. In Malaysia, only dental students have adequate exposure for the dental knowledge and awareness regarding various dental procedures during their graduation program. This clarifies low anxiety among dental students when compared with medical and pharmacy students. Also, there is a lack of dental visits for routine check-up among nondental students in comparison to dental students. Lack of dental visits will further reduce the educational achievements in their studies through their suffering from dental pain when it occurs and avoiding the dental treatment because of their dental fear [18]. Hence, more focus should be made on this aspect to achieve better educational performance and good life style.

In the present study, the mean dental anxiety score declines significantly with each successive year of study. This result is consistent with studies conducted by Peretz and Mann [19], Kirova [20], and Acharya and Sangam [21] which is a direct indication that 1st-year (junior) dental students have greater fear from a dental treatment than 5th-year (senior) dental students. This might be because the students become more aware and more professionally developed and acquire more clinical experience when they advance in their clinical years, and this development is in agreement with the results of study done by Ali et al. [22].

The results of this study showed that the students were more anxious when they probed about how they feel if their tooth is drilled and given local anesthetic injection. Similar findings were also reported in the studies conducted by Hakim and Razak [10], Acharya and Sangam [21], Ali et al. [22], and Humphris and King [23]. The fear of injection is one of the most common hindrances in seeking good medical/dental care. Most likely, this can be because “drill” and “anesthetic needle” are common clinical procedures that can invoke pain. Students in the present study were less fearful towards the nonoperative dental procedures. The lowest mean score was reported for dental appointment. Similar results were found in studies conducted by Hakim and Razak [10] and Domoto et al. [24]. Various dental procedures invoke altered level of fear among individuals. This study suggests that the respondents were more fearful towards the operative procedures than the nonoperative work. The possible reason for this is that the operative dental procedures are directly or bodily applied to them [10].

Thus, further education related to dental health is needed for nondental students to control dental anxiety and improve patient attitudes to dental treatment and compliance. Establishing specialized dental fear clinics may help in managing the anxious patients as well as students to alleviate their dental anxiety. Management of dental anxiety should be taught to dental professionals early as it will reduce the chance of resistant dental anxiety and fear which are difficult to deal with, in the dental clinic [6]. For a successful dental treatment, a supportive and sympathetic approach is desirable. The patient attitudes and behavior towards the dental treatment can be best understood by assessing the anxiety before starting the dental treatment. This information can be utilized in developing the strategies to manage patient anxiety [25].

The limitations of the present study are as follows: Firstly, this is a cross-sectional study and it is important to note that perceptions of dental anxiety can change over time. Thus, the associations identified in such studies should not be considered a causal relationship. Secondly, it is a self-administered questionnaire study where the respondents may hide their true feeling and may also have underreported their dental fear, anxiety, and unpleasantness related to seeking and obtaining dental care.

## 5. Conclusions

Within the limitations of the study it can be concluded that dental students had the lowest dental anxiety scores compared to medical and pharmacy students. Senior dental students with clinical experience showed a lower dental anxiety level than junior dental students. The overall fear of dental treatment was high which necessitates more prevention protocols and inculcation of dental health educational programs in the curriculum. Also, the graded exposure therapy (exposure of clinical procedures) at an initial phase of dental training could help decrease the anxiety levels. Thus, the dental anxiety is one of the constraints in utilization of dental services.

## Figures and Tables

**Table 1:** Distribution of study subjects according to age, gender, and field of study.

Faculty	Age in years (mean ± SD)	Gender	
		Male *n* (%)	Female *n* (%)
Dental (*n* = 308)	22.19 ± 1.65	92 (29.87)	216 (70.13)
Medicine (*n* = 542)	22.21 ± 1.79	207 (38.19)	335 (61.81)
Pharmacy (*n* = 174)	22.06 ± 1.74	43 (24.71)	131 (75.28)

Total (*N* = 1024)	22.15 ± 1.73	342 (33.40)	682 (66.60)

**Table 2:** Distribution of study subjects according to field of study and level of dental anxiety.

Level of anxiety	Faculty			Total *N* (%)	*P* value
	Dental *n* (%)	Medical *n* (%)	Pharmacy *n* (%)		
Low	160 (15.63)	199 (19.43)	56 (5.47)	415 (40.53)	<0.001^*∗∗*(a)^
Moderate	126 (12.30)	237 (23.14)	79 (7.71)	442 (43.16)	
High	22 (2.15)	106 (10.35)	39 (3.81)	167 (16.31)	

Mean score	11.95 ± 4.21	13.80 ± 4.99	14.49 ± 5.09		<0.001^*∗∗*(b)^

Data is presented in proportion and mean ± SD. ^(a)^Chi square test; ^(b)^one-way ANOVA test.

^*∗∗*^ *P* < 0.001, highly significant.

**Table 3:** Year-wise distribution of study subjects by the level of dental anxiety and field of study.

Level of anxiety	Year					Total *n* (%)	*P* value
	First *n* (%)	Second *n* (%)	Third *n* (%)	Fourth *n* (%)	Fifth *n* (%)		
Dental							
Low	23 (7.47)	35 (11.36)	37 (12.01)	34 (11.04)	31 (10.06)	160 (51.95)	0.02^*∗*^
Moderate	36 (11.69)	35 (11.36)	15 (4.87)	24 (7.79)	16 (5.19)	126 (40.91)	
High	6 (1.95)	6 (1.95)	3 (0.97)	6 (1.95)	1 (0.32)	22 (7.14)	
*Total*	*65 (21.10)*	*76 (24.68)*	*55 (17.86)*	*64 (21.10)*	*48 (15.58) *	*308 (100)*	

Medical							
Low	56 (10.33)	38 (7.01)	51 (9.41)	44 (8.12)	10 (1.85)	199 (36.72)	0.28
Moderate	53 (9.78)	41 (7.56)	80 (14.76)	50 (9.23)	13 (2.40)	237 (43.73)	
High	27 (4.98)	17 (3.14)	43 (7.93)	15 (2.77)	4 (0.74)	106 (19.56)	
*Total*	*136 (25.09)*	*96 (17.71)*	*174 (32.10)*	*109 (20.11)*	*27 (4.98)*	*542 (100)*	

Pharmacy							
Low	18 (10.34)	14 (8.05)	12 (6.90)	12 (6.90)		56 (32.18)	0.31
Moderate	19 (10.92)	15 (8.62)	17 (9.77)	28 (16.09)		79 (45.40)	
High	5 (2.87)	11 (6.32)	10 (5.75)	13 (7.47)		39 (22.41)	
*Total*	*42 (24.14)*	*40 (22.99)*	*39 (22.41)*	*53 (30.46 * **)**		*174 (100)*	

Data are presented in proportion and compared by Chi square test. ^*∗*^*P* < 0.05, statistically significant.

**Table 4:** Year-wise comparison of mean anxiety scores as per field of study.

Year	Faculty		
	Dental	Medical	Pharmacy
First	13.05 ± 4.25	13.65 ± 4.83	12.40 ± 4.68
Second	12.38 ± 4.22	13.24 ± 4.79	14.95 ± 5.29
Third	11.11 ± 4.34	14.82 ± 5.20	14.77 ± 5.21
Fourth	12.06 ± 4.11	13.07 ± 4.89	15.58 ± 4.80
Fifth	10.63 ± 3.69	12.93 ± 4.72	
*P* value	0.02^*∗*^	0.02^*∗*^	0.02^*∗*^

Data are presented as mean ± SD and compared by one-way ANOVA test.

^*∗*^ *P* < 0.05, statistically significant.

**Table 5:** Comparison of mean MDAS for each item according to field of study.

Items	Faculty			*P* value
	Dental	Medical	Pharmacy	
If you went to your dentist for treatment tomorrow, how would you feel?	1.87 ± 0.93	2.34 ± 1.21	2.30 ± 1.21	<0.001^*∗∗*^
If you were sitting in the waiting room (waiting for treatment), how would you feel?	2.10 ± 1.01	2.49 ± 1.22	2.54 ± 1.26	<0.001^*∗∗*^
If you were about to have a tooth drilled, how would you feel?	2.79 ± 1.15	3.28 ± 1.23	3.50 ± 1.15	<0.001^*∗∗*^
If you were about to have your teeth scaled and polished, how would you feel?	1.88 ± 1.03	2.45 ± 1.24	2.56 ± 1.32	<0.001^*∗∗*^
If you were about to have a local anesthetic injection in your gum, above an upper back tooth, how would you feel?	3.31 ± 1.19	3.24 ± 1.29	3.58 ± 1.26	0.01^*∗*^

Data are presented as mean ± SD and compared by Kruskal Wallis test. ^*∗*^*P* < 0.05, statistically significant; ^*∗∗*^*P* < 0.001, statistically highly significant.
